# Hybrid Atypical Enteropathogenic and Extraintestinal *Escherichia coli* (aEPEC/ExPEC) BA1250 Strain: A Draft Genome

**DOI:** 10.3390/pathogens10040475

**Published:** 2021-04-14

**Authors:** Danielle D. Munhoz, Fernanda F. Santos, Thais Mitsunari, Paulo A. Schüroff, Waldir P. Elias, Eneas Carvalho, Roxane M. F. Piazza

**Affiliations:** 1 Laboratório de Bacteriologia, Instituto Butantan, São Paulo 05503-900, Brazil; thais.mitsunari@butantan.gov.br (T.M.); paulo.schuroff@butantan.gov.br (P.A.S.); waldir.elias@butantan.gov.br (W.P.E.); eneas.carvalho@butantan.gov.br (E.C.); 2 Departamento de Microbiologia, Imunologia e Parasitologia, Escola Paulista de Medicina, Universidade Federal de São Paulo, São Paulo 04023-062, Brazil; ff.santos@unifesp.br

**Keywords:** *E. coli*, diarrhea, aEPEC, genome evolution, extraintestinal pathogen

## Abstract

Diarrheagenic *Escherichia coli* is the major bacterial etiological agent of severe diarrhea and a major concern of public health. These pathogens have acquired genetic characteristics from other pathotypes, leading to unusual and singular genetic combinations, known as hybrid strains and may be more virulent due to a set of virulence factors from more than one pathotype. One of the possible combinations is with extraintestinal *E. coli* (ExPEC), a leading cause of urinary tract infection, often lethal after entering the bloodstream and atypical enteropathogenic *E. coli* (aEPEC), responsible for death of thousands of people every year, mainly children under five years old. Here we report the draft genome of a strain originally classified as aEPEC (BA1250) isolated from feces of a child with acute diarrhea. Phylogenetic analysis indicates that BA1250 genome content is genetically closer to *E. coli* strains that cause extraintestinal infections, other than intestinal infections. A deeper analysis showed that in fact this is a hybrid strain, due to the presence of a set of genes typically characteristic of ExPEC. These genomic findings expand our knowledge about aEPEC heterogeneity allowing further studies concerning *E. coli* pathogenicity and may be a source for future comparative studies, virulence characteristics, and evolutionary biology.

## 1. Introduction

Diarrhea is the second leading cause of death in children under five years of age, leading approximately 525,000 children to death annually. In low-income countries, children under three years old experience an average of three episodes of serious diarrhea each year, resulting in severe cases of malnutrition and death [1]. Infections of the intestinal tract usually have diarrhea as one of the symptoms and can be caused by a wide variety of bacteria, viruses, and parasites [2], and the main bacterial agent are diarrheagenic *Escherichia coli* (DEC) strains. The frequency of infections caused by these microorganisms can be even higher since in 34% of diarrhea cases the causative agent is not identified [3].

Diarrheagenic *E. coli* evolved by acquiring specific set of characteristics by horizontal gene transfer. The current DEC pathotypes are classified according to the set of acquired genes, and differ in terms of virulence factors, mechanisms of pathogenicity and clinical symptoms [4,5,6]. Despite the presence of specific virulence factors in each pathogroup, *E. coli* has great genomic plasticity, which has led to the identification of isolates with a combination of virulence characteristics from different pathotypes. These isolates are considered potentially more virulent and are called hybrid pathogenic strains [4,7], as the strain responsible for a large diarrhea outbreak in Germany in 2011 [8,9,10].

Enteropathogenic *E. coli* (EPEC) is highly prevalent in community settings such as schools and hospitals and is a major cause of childhood diarrhea in developing country. It is estimated that EPEC is responsible for 5–10% of all cases of pediatric diarrhea in countries like Brazil, Chile, Peru, and Iran [11] and, in addition to colonizing humans, this pathogen is also capable of infecting animals such as cattle, dogs, cats, wild boar, and rabbits [12,13,14,15]. EPEC is subdivided into typical EPEC (tEPEC) and atypical EPEC (aEPEC), based on the presence of the EPEC adherence factor (EAF) plasmid in the former group and its absence in the latter group [16]. In addition, aEPEC neither express nor produce the bundle-forming pilus (BFP), a type four fimbriae [17,18,19].

Atypical EPEC strains have been described as an important agent of diarrhea since the 1990s and are currently frequently detected in underdeveloped countries [3,20]. Unlike tEPEC, aEPEC has been detected in patients of all ages and also in immunosuppressed individuals [21,22,23,24]. This is an extremely heterogeneous group in terms of virulence factors [25,26], genetic characteristics, in vitro adhesion phenotype, O:H serotypes [27,28], and host type and immunological condition [12,25]. Previous genome assemblies showed that the genome of some aEPEC strains contains different DEC and extraintestinal pathogenic *E. coli* (ExPEC) genetic characteristics [29,30,31], indicating that this pathotype genome had undergone unique evolutionary events.

In this work, we report the sequencing, assembly, and annotation of the genome of a strain previously classified as aEPEC, BA1250 [17], according to this pathotype genetic markers, as the presence of genes that comprise the pathogenicity island called Locus of enterocyte effacement (LEE) [32]. Paired-end sequencing and trimming of the adapter and small or low-quality sequences were performed. The assembly and annotation of the genome were carried out using the SPAdes and Prokka pipelines, respectively. Interestingly, phylogenetic analysis showed a more recent relationship among BA1250 with *E. coli* strains responsible for urinary infection than with other aEPEC strains. In addition, this strain genome presents a set of genes characteristic of ExPEC, which allows its classification as a hybrid strain.

The genome of BA1250 aEPEC/ExPEC strain will be useful for comparative studies as a genomic reference to allow analyses of singular virulence factors and for studies of molecular and evolutionary biology.

## 2. Results and Discussion

### 2.1. Overview of BA1250 Draft Genome Assembly

Approximately 3.6 million fragments of genomic DNA (reads) were sequenced, totalizing approximately 915 million sequenced bases. From this total, adapters sequence, low-quality bases, small reads, and unmatched reads were removed, resulting in approximately 3.4 million reads and 400 million bases, a reduction of approximately 6.9% and 56.9% in the total sequenced, respectively. This value represents the coverage of approximately 77 times, considering an average *E. coli* genome size of 5.1 million bases. The sequencing performed generated data that allow the assembly of a representative genome, as coverage of 30 times is already considered sufficient [33].

The assembled genome has 4,964,536 bases and assembly resulted in 151 contigs, with an N50 of 129,195 bases, and 4717 genes identified after annotation. There were 1261 genes that overlap another gene on the same strand. Comparing to the genome of the prototype tEPEC strain E2348/69 (GCA_000026545.1), there were 83.14% of aligned regions and 79.14% of the genes of the reference strain were found in BA1250. These results indicate that, although it has its particularities, the assembled genome is similar to a reference genome, which suggests that the sequencing and the assembly step could generate material with a good representation of the genomic content of the BA1250 strain. The overall BA1250 draft genome feature (including chromosome and plasmid content) is shown in Table 1.

### 2.2. Plasmid Content

The results obtained by Illumina sequencing (Illumina, Inc., San Diego, CA, USA) and the SCAPP [34] assembler allowed the identification of a complete plasmid sequence. The plasmid, assembled to a unique contig, has 154,710 base pairs, with 158 CDS and 158 genes identified after annotation carried out using the Prokka pipeline.

Plasmid classification into Incompatibility (Inc) groups is based on the sequence of the replication initiation (Rep) protein. Using Plasmid Finder software [35], we have identified, with 96,5% of identity, the Inc group IncFIB/IncFII in the assembled plasmid. Plasmids classified as IncFIB/IncFII are found in bacteria from different animals and human hosts, and the strains belong to several phylogroup, as A, B1, B2, D, and E [36]. The F plasmid is a primary example of a large group of conjugative plasmids, widespread in *E. coli.* It is worth mentioning that virulence-associated traits of *E. coli* are almost exclusively found on IncF-family plasmids, making these plasmids of great clinical relevance [37,38]. The majority of proteins comprised in F-family plasmid-mediated bacterial conjugation are expressed from a polycistronic *tra* operon in the plasmid transfer (*tra*) region that contains basically the same organization of conjugation-related genes [39,40]. In the BA1250 plasmid sequence, based on an additional analysis carried out with Abricate [41] were identified nine *tra* genes, as *traA*, *traC*, *traD*, *traJ*, *traM*, *traQ*, *traS*, *traV*, and *traY*.

Among the 158 genes present in this plasmid, is the *vat* gene, a highly prevalent and tightly regulated immunogenic serine protease autotransporter protein of Enterobacteriaceae (SPATE) usually secreted by ExPEC during infection and considered as an ExPEC marker [42,43,44]. It is also present in the plasmid sequence, seven genes of the *fae* operon, which encodes subunits of chaperone-usher assembled family K88 fimbriae [45].

### 2.3. Phylogenetic Analyses

**Figure 1:**
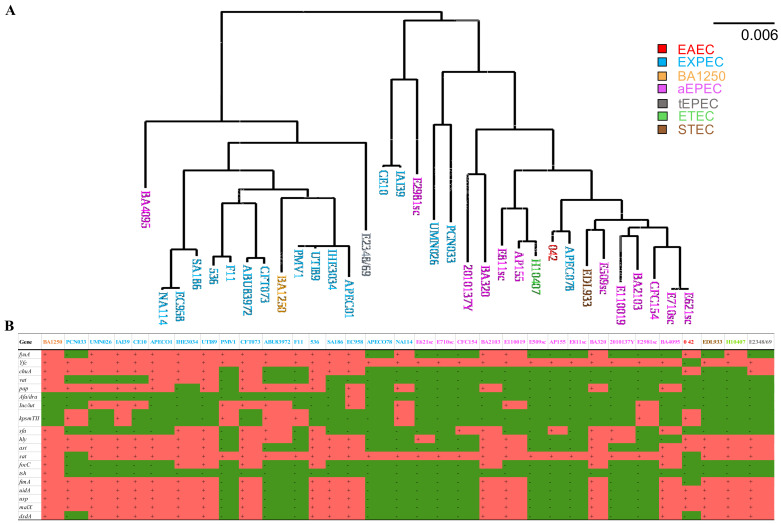
BA1250 phylogenetic analysis. (**A**) The phylogenetic tree was built with 13 atypical enteropathogenic *E. coli* (aEPEC) strains, 4 diarrheagenic *Escherichia coli* (DEC) prototypes, and 16 extraintestinal pathogenic *E. coli* (ExPEC) strains genome. Strains are colored according to pathogenic *E. coli* group, as blue for ExPEC strains, brown for Shiga-toxin-producing *E. coli* (STEC) prototype strain EDL933, pink for aEPEC strains, red for enteroaggregative *E. coli* (EAEC) prototype strain 042, gray for typical enteropathogenic *E. coli* (tEPEC) prototype strain E2348/69, green for enterotoxigenic *E. coli* (ETEC) prototype strain H10407 and orange for the aEPEC strain BA1250. (**B**) Specific ExPEC gene survey among selected ExPEC, aEPEC and prototypes DEC strains. All strains names are colored as described for phylogenetic tree. Red squares represent presence of gene and Green squares represent its absence.

To understand the general genomic relationship of BA1250 with other *E. coli* isolates, phylogenetic analyses were performed based on the genome of 13 aEPEC strains, 4 DEC prototypes, and 16 ExPEC strains. Phylogeny tree indicated that BA1250 shares a more recent common ancestor with *E. coli* strains isolated from extraintestinal infections than with other aEPEC strains (Figure 1A). In addition to the completely different site of isolation, this analysis demonstrates that BA1250 and ExPEC share genetic characteristics and are more closely related to each other than BA1250 with the other strains of aEPEC selected for the analysis.

Hybrid *E. coli* pathotypes are being classified as emerging public health threats with enhanced virulence due to genetic features from different pathotypes. To determine whether BA1250 could be classified as a hybrid strain, a deeper genetic survey was performed. Most of DEC pathotypes possess a set of characteristic genes that are targeted in pathotype classification, epidemiologic studies as well as in diagnostic tools. Although their isolation site designates ExPEC strains, some groups of genes are often employed as ExPEC markers, since it does not have exclusive and well-defined core of characteristic genes. Research groups use distinct set of genes, usually four or five genes, to study virulence factor among ExPEC strains [29,42,43,44]. In this study we have selected 19 genes of the most common virulence features of this pathotype to investigate whether BA1250 may be classified as a hybrid strain. A survey in BA1250 genome revealed that, in fact, this is a hybrid strain, since it possesses 16 genes that are ExPEC markers (Figure 1B).

**Figure 2:**
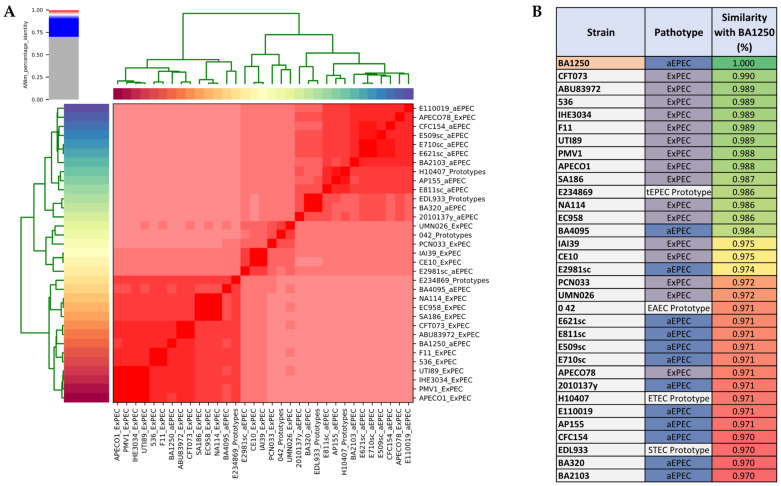
Comparison of Average Nucleotide Identity between each of the 33 strains used in this study. (**A**). The cells in the heatmap are colored according to an Average Nucleotide Identity (ANI) value demonstrated at the top left box. The dendrograms (above and on the left side), correspond to the results of the clustering of the ANI values between the used strains. (**B**). The figure demonstrates the percentage of similarity specifically with BA1250, colored according to a colour scale from green to red.

Considering that BA1250 has the genetic markers of ExPEC and may now be classified as hybrid strain, we applied the Average Nucleotide Identity (ANI) [46] and compared its genome with the 16 ExPEC, 12 aEPEC and 4 DEC prototypes strains used in the phylogenetic tree. As presumed, the comparative assay revealed a high similarity between each strain from a specific pathotype, as aEPEC and ExPEC, as depicted by the hierarchical clustering based on ANI values (Figure 2A). Likewise, there were reduced similarities between the strains from different pathotypes. In accordance with the phylogenetic analysis, BA1250 has more similarity with the ExPEC group, showing a higher level of identity with the extraintestinal pathogen compared to the intestinal pathogen (Figure 2B).

Unlike BA1250, the most notorious hybrid strain was originated from two DEC pathotypes, instead of DEC and ExPEC. This Shiga-toxin-producing *E. coli* (STEC)/enteroaggregative *E. coli* (EAEC) strain was responsible for a large outbreak with numerous hemolytic-uremic syndrome (HUS) cases and deaths in Germany in 2011 [10]. The strain was initially classified as STEC due to the presence of *stx* genes encoding Shiga toxin, but were notably more virulent, since it led to a higher frequency of HUS cases and even deaths. Genome sequencing and phylogeny analysis revealed a significant genetic similarity with EAEC strains and the presence of classical EAEC virulence genes as *aggA* and *aggR*.

Some DEC/ExPEC hybrid strains also have been identified, for instance the STEC/ExPEC O80:H2 hybrid reported to cause HUS and bacteremia [47]. This specific strain was successively isolated from the patient stool and blood sample, showing its extreme and diverse virulence capacity. Genetic analysis revealed the presence of the typical STEC *stx* genes and some ExPEC genes like *iucC* and *hlyF* [48]. Similarly, a significant number of strains were isolated from hospitalized patients and further characterized according the genetic and phenotypic characteristics in in vitro assays as DEC/ExPEC hybrids [49].

Concerning another EPEC/ExPEC hybrid, isolated from a case of a patient with severe protracted diarrhea, bacteremia and multiorgan dysfunction, genome sequencing revealed that this infection was caused by an ExPEC strain that also featured distant orthologs of genes characteristic of EPEC [50]. This strain displayed some characteristic of ExPEC, including complete sets of genes for P fimbriae, hemolysin, and the *kpsM* gene used to identify group II capsule loci [51]. The study also revealed that this isolate has the genetic markers for EPEC, including the presence of LEE genes and the absence of *stx1* and *stx2* genes encoding Shiga toxins, which are found in addition to the LEE in enterohemorrhagic *E. coli* (EHEC) strains [16]. However, some essential LEE genes were not found or displayed low similarity, raising doubt as to whether the characteristic EPEC Type 3 secretion system (T3SS) is fully functional in this isolate.

### 2.4. BA1250: A Hybrid Strain

The ability of a specific strain to cause disease relay in a specific combination of genes encoding virulence factors. The core genome shared by *E. coli* strains corresponds to approximately 40% of its average 5000 genes, while the total number of genes that exist in all *E. coli* strains is predicted to exceed 15,000 [52,53]. Each diarrheal and extraintestinal *E. coli* pathotype possess a distinguished genomic feature that illustrates the phenotypical characteristics.

In this study we described a number of genomic features from BA1250, a strain that were initially classified as aEPEC. This classification was based on the EPEC unique characteristic that is the presence of a pathogenicity island named as Locus of Enterocyte Effacement (LEE) [32]. This pathogenicity island is composed of 41 genes and some of them are used as representative gene markers for EPEC, such as *eae*, encoding the adhesin intimin, and *tir*, encoding the intimin translocated receptor. This pathogenicity island is likely to encode almost all of the genes necessary to produce an intestinal attaching/effacing (A/E) lesion, the histopathological lesion characteristic of EPEC infection. In addition to *eae* and *tir*, LEE comprises genes that include a type III secretion system (TTSS), several secreted proteins (*espB*, *espD*, and *espF*), and their chaperones [54,55,56]. Distinct from EPEC, a strain is usually classified as ExPEC according to its extraintestinal site of isolation. While several virulence factors are associated with ExPEC, until now there is no core set of genes that can be used to definitively differentiate these pathotypes. However, over the last years, authors have observed some group of genes more frequently identified among ExPEC strains, thus being applied as genetic markers [29,42,43,44,57]. These set of genes vary among four or five genes, such as *fyuA*, *yfc*, *chuA*, and *vat*, which, among others, were identified in the BA1250 genome, allowing its classification as a hybrid strain.

**Figure 3:**
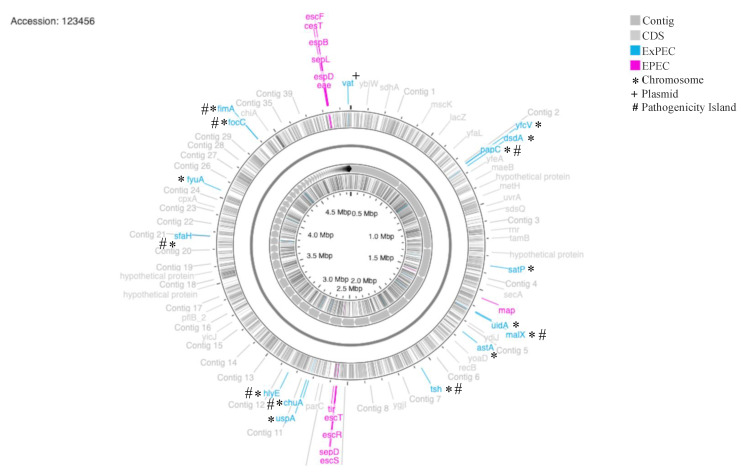
Illustrative BA1250 draft genome (including chromosome and plasmid contents). Representative LEE genes from EPEC are stained in pink. The selected ExPEC genes for the study are stained in blue. * genes present in chromosome, † gene present in plasmid and # genes that are part of pathogenicity islands. Contigs are represented as gray arrows in the inner circle and are classified with respect to their size. Colored lines represent genes in the positive and negative-sense DNA strand into the outer and inner circle, respectively.

*E. coli* is a highly diverse group, yet still closely related microorganism. Until now, there is not a consensus regarding the exact evolution process of DEC strains [58,59]; thus, it is still not feasible to classify which part of genome belongs to each pathotype. Nevertheless, as we mentioned, some groups of genes are described for each pathotype and applied as genetic marker, as LEE genes are for EPEC and 19 genes of ExPEC selected for this study. In Figure 3 we highlighted some of the LEE genes and ExPEC genome block in BA1250 genome. All LEE genes are grouped as a pathogenic island present in the EPEC strains chromosome (revised in 25). Except from *vat* gene that were found in the BA1250 plasmid, all other 15 ExPEC genes, were also identified in the chromosome, where genes *fimA*, *focC*, *sfaH*, *hlyE*, *chuA*, *tsh*, *malX*, and *papC* are part of different pathogenicity islands according to literature and predicted by Island Viewer 4 [60,61,62].

Considering the emergence and public health significance of hybrid pathotypes, this new pathogenic group should be studied in closer details to better understand the hybrid infection. The genome of BA1250 is a very interesting model of a hybrid strain and this sequencing investigation allows future comparison analyzes.

## 3. Materials and Methods

### 3.1. Strain Isolation and Identification

BA1250 strain analyzed in this study was isolated from feces in a case-control epidemiologic survey from children with diarrhea in Salvador, Brazil. The strain was classified as atypical enteropathogenic *E. coli* (aEPEC, *eae*+/EAF−/*stx*−/BFP−), displaying the localized adherence-like (LAL) pattern on HEp-2 cells [17,63]. The strain was stored at −80 °C in Luria Bertani broth (LB) with 25% glycerol and was routinely grown in LB for 18 h at 37 °C.

### 3.2. DNA Extraction, Sequencing Library, and Read Filtering

For BA1250 genome sequencing, the strain was grown in LB broth for 18 h at 37 °C, and DNA was extracted using the Wizard^®^ Genomic DNA Purification Kit (Promega, Madison, WI, USA), according to the manufacturer’s protocol. The sequencing was performed using Illumina technology on the Illumina Hiseq1500 platform, with kits from the same company and following the manufacture’s guidelines. The genomic library was prepared using the HiSeq Rapid SBS Kit v2 library kit, and paired-end sequencing was performed with 2 × 250 sequencing cycles. The resulted sequences were treated for the removal of adapter, low-quality bases, and small reads, using the Trimmomatic tool [64].

### 3.3. Genome Assembly and Annotation

Paired-end reads were assembled using SPAdes version 3.11.1 [65] and QUAST tool was applied to analyze the assembled genome content and align it to a reference genome E2348/69 (GCA_000026545.1) [66]. Finally, the assembled genome was subjected to automated annotation by Prokka program version 1,12 [67]. The whole-genome sequences (WGS) were deposited in the GenBank database under the accession number JADPBX000000000, BioProject and SRA data PRJNA678986.

### 3.4. Plasmid Identification

Reads were assembled using SCAPP [34] and PlamisFinder tool [35] was applied to analyze the assembled genome content. The assembled plasmid was subjected to automated annotation by Prokka program version 1,12 [67] that enabled protein identification.

### 3.5. Phylogenetic Analysis

The assembled genome from BA1250 was submitted to the comprehensive genome analysis service at Pathosystems Resource Integration Center (PATRIC) [68], where a phylogenetic analysis was carried out, on the “Phylogenetic Tree Building” using default option “Codon Tree”. Phylogeny tree were edit with TreeDyn 198.3 at Methods et algorithmes pour la bio-informatique from LIRMM [69,70,71]. The analysis was performed based on the genome of 13 aEPEC strains (BA1250; Strain 2010137Y; AP155 (HE-8); E110019; BA4095; BA2103; BA320; E710sc; E621sc; E811sc; E2981sc; E509sc; CFC_154), 4 DEC prototypes (ETEC strain H10407; EHEC strain EDL933; EAEC strain 042; tEPEC strain E2348/69) and 16 ExPEC strains (APEC078; PCN033; UMN026; IAI39; CE10; APEC01; IHE3034; UTI89; PMV1; CFT073; ABU83972; F11; 536; SA186; EC958; NA114). All aEPEC strains were isolated from human samples, except strain AP155 (HE-8) that was isolated from dog and strain CFC_154 that was isolated from cow. Gene specific analyses were carried out with Sublime Text editor.

The different genome comparison was carried out with Average Nucleotide Identity (ANI), using the software pyany [46]. This is a category of computational analysis that usually involves the fragmentation of genome sequences, followed by nucleotide sequence search, alignment, and identity calculation.

### 3.6. Pathogenic Island Predictiom

The assembled genome from BA1250 was submitted to the server of Island Viewer [63]. IslandViewer is a computational tool that integrates four different genomic island prediction methods: IslandPick, IslandPath-DIMOB, SIGI-HMM, and Islander.

## 4. Conclusions

This study clearly demonstrated that the genetic background of BA1250 closely resembled to ExPEC strains, which could indicate that this is a hybrid strain and may be of greater virulence compared to other aEPEC, since it possesses machinery to possibly infect different niches other than the intestine. This comparative analysis provided a comprehensive understanding of an aEPEC strain genome and closely related species that allow future studies on hybrid strains.

## Figures and Tables

**Table 1:** Summary statics of BA1250 draft genome assemblies.

Feature	Value
Contigs	151
GC Content (%)	50.56
Contig L50	9
Contig N50	129,195
Genome length (bp)	4,964,536
Protein coding sequences (CDS)	4969
Annotated genes	4717
Transfer RNA (tRNA)	87
Ribosomal RNA (rRNA)	12

## Data Availability

The data underlying this article are available in the GenBank database under the accession number JADPBX000000000, BioProject and SRA data PRJNA678986, at https://www.ncbi.nlm.nih.gov/genbank/ (accessed on 15 January 2021).
